# Supplementary material for: Chromothripsis during telomere crisis is independent of NHEJ, and consistent with a replicative origin
Source: Genome Res. 2019 May;29(5):737–49. doi: 10.1101/gr.240705.118 (PMC6499312; doi:10.1101/gr.240705.118)
Supplement: Supplemental Material [file supp_gr.240705.118_Supplemental_file_1.zip › contigs/annotated_contigs/DB54/contig.2.DB54_length_345_mean_cov_6.07826086957.docx]

**DB54_length_345_mean_cov_6.07826086957**

```
TCCACCTGCCTCGGTCTCCCAAAGTGGTAGGATTACAGGCGTGAGCCACCATGCCTGGGCTGGCCTGTTGTATCTTATGCTCATCGCAG
 >chr5:60950513-60950781 + E=3e-147
CCTTCCCCCTGCCCTGGTCCTCAGGCATCCCTGCAGGCTTTTGCTCAGCTAGCTGCTGAGTCATGTGGTCTGTGAAGACTGGAGAGCTG

CAGGAGAAAAGAAGCCCTGAGAGTCCTCACCTTCTCTTTCTGCTTTGGCAGCATCACCAGCTGTGGCTGGGTCTTTTTTCTGGACTCCA

G|AAAAAC|ATTCTAACACCAGTTAGAATGGCAGTCATTAAAAAGTCAGGAAACAACAGGTGCTGGAGAGGATGTGGAGA        >chr6:122230457-122230528 - E=3e-28
```
